# Defining the Signature of VISTA on Myeloid Cell Chemokine Responsiveness

**DOI:** 10.3389/fimmu.2019.02641

**Published:** 2019-11-19

**Authors:** Thomas W. K. Broughton, Mohamed A. ElTanbouly, Evelien Schaafsma, Jie Deng, Aurélien Sarde, Walburga Croteau, Jiannan Li, Elizabeth C. Nowak, Rodwell Mabaera, Nicole C. Smits, Anna Kuta, Randolph J. Noelle, J. Louise Lines

**Affiliations:** ^1^Department of Microbiology and Immunology, Norris Cotton Cancer Center, Geisel School of Medicine at Dartmouth, Lebanon, NH, United States; ^2^Division of Transplantation Immunology & Mucosal Biology, Faculty of Life Sciences & Medicine, King's College London, London, United Kingdom; ^3^Section of Hematology and Oncology, Dartmouth-Hitchcock Medical Center, Lebanon, NH, United States; ^4^Immunext Corp., Lebanon, NH, United States

**Keywords:** VISTA, macrophage, immune checkpoint, chemokine, migration

## Abstract

The role of negative checkpoint regulators (NCRs) in human health and disease cannot be overstated. V-domain Ig-containing Suppressor of T-cell Activation (VISTA) is an Ig superfamily protein predominantly expressed within the hematopoietic compartment and has been studied for its role in the negative regulation of T cell responses. The findings presented in this study show that, unlike all other NCRs, VISTA deficiency dramatically impacts on macrophage cytokine and chemokine production, as well as the chemotactic response of VISTA-deficient macrophages. A select group of inflammatory chemokines, including CCL2, CCL3, CCL4, and CCL5, was strikingly elevated in culture supernatants from VISTA KO macrophages. VISTA deficiency also altered chemokine receptor recycling and profoundly disrupted myeloid chemotaxis. The impact of VISTA deficiency on chemotaxis *in vivo* was apparent with the reduced ability of both KO macrophages and MDSCs to migrate to the tumor microenvironment. This is the first demonstration of an NCR impacting on myeloid mediator production and chemotaxis, and will guide the use of anti-VISTA therapeutics to manipulate the chemotaxis of inflammatory macrophages or immunosuppressive MDSCs in inflammatory diseases and cancer.

## Introduction

Chemokine/cytokine production and chemoattractant-mediated migration of immune cells is critical for immune surveillance and homeostasis. Chemokines play integral roles in the spatio-temporal coordination of immune cell migration and positioning (1). Chemokines control the migratory behavior, positioning, and important interactions between cell subsets; all critical activities for an intact immune response (2). Upon activation, myeloid cells undergo a distinctive signature of chemokine/cytokine and chemokine/cytokine receptor expression changes (3–5) that uniquely prime them for a productive immune response.

V-domain Ig-containing Suppressor of T-cell Activation (VISTA, also known as VSIR, PD-1H, Dies1, DD1α, and GI24) is a type I transmembrane protein and member of the immunoglobulin superfamily (IgSF) with homology to the Programmed Death-1 (PD-1) family of proteins. VISTA is largely expressed within the hematopoietic compartment with expression on naïve CD4^+^ and CD8^+^ T cells and higher expression on macrophages, patrolling and inflammatory monocytes, neutrophils, and both lymphoid and myeloid subsets of dendritic cells (6–8). Initial insights into a potential role of VISTA in myeloid biology was based on the observation that VISTA deficiency attenuated collagen antibody-induced arthritis (CAIA) (9). Although the VISTA deficient myeloid cells showed an enhanced inflammatory phenotype in that model, a significant reduction of C5aR expression on monocytes and macrophages was observed. The reduced expression of C5aR correlated with reduced migration and signaling responses to C5a. Since the C5a/C5aR pathway is critical for CAIA development (10–12), this provided rationale for the reduced disease incidence in VISTA deficient mice.

Beyond its impact on chemotaxis, data is also emerging that VISTA impacts on multiple aspects of myeloid biology. One study showed that enforced overexpression of VISTA on monocytes induced elevated levels of cytokine expression (13). Furthermore, anti-VISTA has been shown to mitigate the suppressive activity of myeloid-derived suppressor cells (MDSCs) (14). Finally, VISTA was shown to play a role in efferocytosis by myeloid cells (15). Taken together, these studies warranted a more detailed analysis of the intrinsic role of VISTA on myeloid biology.

The studies presented herein focus on the impact of the genetic loss of VISTA on myeloid chemotaxis and chemokine/cytokine production. We show that VISTA deficiency disrupts chemokine receptor recycling, chemokine consumption, and results in heightened culture chemokine levels which is not dependent on enhanced transcription. Strikingly, the chemotactic responses of VISTA deficient myeloid cells to a select group of inflammatory chemokines is profoundly impaired. The chemotactic paralysis of VISTA deficient myeloid cells is evident both *in vitro* and *in vivo*. This is the first description of a negative checkpoint regulator (NCR) playing a central role in myeloid chemokine production and chemotaxis, and underscores its important function in controlling both innate and adaptive immunity.

## Materials and Methods

### Mice

Eight to ten week-old female C57BL/6 mice (WT) and BALB/cAnNCrl (BALB/c) were purchased from Charles River (Wilmington, MA). B6N.129S5(B6)-*Vsir*^*tm*1*Lex*^/Mmucd (VISTA KO) mice were obtained from the Mutant Mouse Regional Resource Centers (www.mmrrc.org; stock no. 031656-UCD) and were fully backcrossed onto the C57BL/6 and BALB/c backgrounds. VISTA^fl/fl^ mice were generously provided by Sam W. Lee. Generation and screening of VISTA^fl/fl^ mice was described previously (15). Conditional deletion of VISTA in the myeloid compartment was achieved by crossing VISTA^fl/fl^ mice to hemizygous B6J.B6N(Cg)-*Cx3cr1*^*tm*1.1(*cre*)*Jung*^/J mice. Cre positive mice were compared to Cre negative littermate controls. Deletion of VISTA in the myeloid compartment was confirmed by flow cytometry as described below. All animals were bred and maintained in a pathogen-free facility at the Geisel School of Medicine at Dartmouth College, NH, USA.

### Tumor Growth

Cells were cultured in RPMI 1640 medium supplemented with 10% FBS, 100 U/ml penicillin, 0.1 mg/ml streptomycin, and 2 mM L-glutamine. CT26.WT colon carcinoma cells were purchased from the American Type Culture Collection (Product: ATCC CRL-2638, Lot Number: 63226308) and experiments used vials frozen from the third passage. Tumor inoculations were performed intradermally (i.d.) into the flank of WT and VISTA KO mice of BALB/c (CT26) backgrounds, with 10^5^ cells per mouse. Tumors were measured three times a week with digital calipers and volumes were determined using the formula: volume = 0.5 × length × width^2^. Mice were euthanized if they showed any sign of morbidity or if tumors exceeded 15 mm in any direction.

### Isolation of Immune Cells

Bone marrow (BM) cells were flushed from the femurs and tibia. BM-derived macrophages (BMDMs) were generated by culture of BM cells in 20 ng/ml recombinant M-CSF (PeproTech, Rocky Hill, NJ, USA) for 7 days (16, 17). Splenocytes were isolated by homogenizing spleens through 40 μm cell strainers (Becton Dickinson, Franklin Lakes, NJ). Red blood cells were lysed using a Tris-buffered Ammonium Chloride buffer.

Peritoneal macrophages were obtained by i.p. injection of WT and VISTA KO mice with 10% (w/v) of Brewer's modified thioglycolate medium (Becton Dickinson) and subsequent peritoneal lavage 4 days post-injection (17, 18).

MDSCs were isolated from spleens of mice bearing size-matched tumors at 14 d post-tumor inoculation using the Myeloid-Derived Suppressor Cell Isolation Kit from Miltenyi Biotech according to the manufacturer's instructions (Miltenyi Biotech, Bergish Gladbach, Germany).

Immune cells infiltrating tumors were isolated using the Miltenyi Tumor Dissociation kit according to the manufacturer's instructions.

### Flow Cytometry and Analysis

Cells prepared as described above were stained for flow cytometry in V-bottom plates (Thermofisher, Waltham, MA). Dead cells were labeled with Live/Dead Fixable Yellow or Near-IR dead cell stain (Invitrogen) in PBS. Surface staining was carried out in PBS containing 0.1% BSA and 5 μg/ml of anti-CD16/32 (Fc block; Thermofisher) for 20 min on ice. For differential staining of mature myeloid cell populations, antibodies used were Ly6C A488, Ly6G APC, F4/80 APC/Cy7 (all from Biolegend, San Diego USA), and CD11b e450 (Thermofisher). To confirm VISTA deletion, cells were stained with VISTA APC (MIH63, Biolegend). Chemokine receptors were stained with CCR3 PE-Cy7 (Biolegend), CCR5 PE (Thermofisher), CCR1 PE, and CCR2 APC (R&D systems, Minneapolis, MN). For BM precursor analysis, cells were stained with antibodies from Biolegend: c-kit FITC, CD115 PE, Ly6C APC-Cy7, CX_3_CR1 PE-Cy7, and a lineage dump in BV421 (TER119, CD3, B220, CD11b, CD11c). Cells were washed in PBS, and then fixed for 20 min at room temperature (RT) in methanol-free formalin before being returned to PBS.

For intracellular cytokine staining, surface staining was performed as above, except Brefeldin A (BioLegend) was included during any stimulations and in all solutions prior to fixation. Cells were then permeabilized and stained intracellularly using BD Perm Buffer (Becton Dickinson). First, cells were pre-blocked with 5 μg/ml of anti-CD16/32 for 10 min at RT, then without washing, anti-CCL3-PE (DNT3CC, Thermofisher) was added for at least 30 min. Cells were washed twice in BD Perm buffer, and then transferred to PBS.

Stained cells were filtered through 60 μm nylon mesh and flow cytometric data was acquired on a MACSQuant Analyzer 10 (Miltenyi Biotec) and analyzed using either FlowJo software (Treestar Incorporated, Ashland, OR) or FlowLogic software (Inivai technologies, Victoria, Australia).

### Stimulation of Macrophages With Innate Immune Ligands

BMDMs and thioglycolate-induced peritoneal macrophages were seeded in flat-bottom 24-well Corning Costar plates (Corning, NY, USA) at a density of 1 million cells/ml and incubated overnight to permit reattachment. Cells were then washed and given fresh medium containing ultra-purified LPS from *Escherichia coli* K12 (Ultrapure LPS-EK), IFNγ, Beta-1,3-glucan from *Alcaligenes faecalis* (Curdlan AL), or poly(deoxyadenylic-deoxythymidylic) acid sodium salt poly(dA:dT). TLR ligands were obtained from InvivoGen (San Diego, CA, USA). Cytokines and chemokines used in these studies were obtained from PeproTech. Experiments were performed with varying concentrations of ligand, but data is presented from the dose that gave the optimal response in WT controls.

### Cytokine Analysis

Simultaneous determination of multiple cytokine concentrations was carried out using the Bio-Plex Pro Mouse Cytokine 23-plex Assay (BioRad Laboratories, Hercules, CA) or the MILLIPLEX MAP Mouse Cytokine/Chemokine Magnetic Bead Panel—Premixed 32 Plex (EMD Millipore, Billerica, MA) on a Bio-Rad Bio-Plex Array Reader. Where repeats of a given experiment (for example, LPS stimulation of BMDMs) were performed, these were run on Luminex kits from the same batch. The following cytokines were assayed for by ELISA: IFNβ (BioLegend), CCL3 (ThermoFisher), and CCL2 (ThermoFisher). All kits were used according to the manufacturer's instructions. Samples were diluted in cell culture medium to the dynamic range of each kit.

### Bone Marrow CFU Analysis

Mouse bone marrow progenitors were isolated from whole bone marrow using the EasySep Mouse Hematopoietic Progenitor Cell Isolation Kit (Stemcell Technologies, Cambridge, MA) per manufacturer's instructions. Colony forming unit (CFU) assays were performed by plating 10^4^ cells in 35-mm culture dish in MethoCult GF M3434 medium (Stemcell Technologies). Colony counts were performed after 12 days of culture and the average of triplicate cultures per bone marrow was recorded.

### Gene Expression Analysis

RNA was isolated from BMDMs using Qiagen RNeasy kits according to the manufacturer's protocol. Cells were processed from 3 to 4 individual mice, with two technical replicate RNA samples per mouse. For Nanostring, RNA samples were analyzed by gene expression analysis and quantified with the Digital Analyzer (NanoString Technologies, Seattle, WA, USA). Expression of 770 genes were analyzed using nCounter Myeloid Innate Immunity Panel. For quantitative RT-PCR, RNA was processed with the Qiagen RNAse-Free DNAse Set 100, and cDNA was generated using the iScript cDNA Synthesis Kit (BioRad Laboratories) according to the manufacturer's instructions. Quantitative PCR was performed using the iCycler thermal cycler (BioRad) fitted with a MyiQ optical module (BioRad). *Actb* was used as an internal control. Real-time primer sequences were *ccl3*: forward: GCGGCTGATGATTGGACAA, reverse: ATCTCCAGCTCGAGCAATGG and *actb* forward: GACCTCTATGCCAACACAGT, reverse: AGTACTTGCGCTCAGGAGGA.

### Single Cell RNA Sequencing and Analysis

Monocytes from CX_3_CR1-Cre VISTA KO vs. WT mice were FACS-sorted on a BD FACS ARIA II cell sorter, with CD11b^+^ sorted and all other populations excluded via lineage gating (CD11c, CD4, CD8, NK1.1, Dead fixable dye, CD19, B220). Droplet-based 5′ end single-cell RNA sequencing (scRNAseq) was performed by the 10x Genomics platform and libraries were prepared by the Chromium Single Cell 5' Reagent kit according to the manufacturer's protocol (10x Genomics, CA, USA). The Cell Ranger Single-Cell Software Suite (10x Genomics) was used to perform barcode processing and transcript counting after alignment to the mm 10 reference genome with default parameters.

Low-quality cells were discarded if mitochondrial gene expression was larger than 10%, <500 genes were detected, or <1,000 total reads were detected. Only genes detected in at least one cell were kept in the count matrix. Library normalization was performed by the *calculateCPM* function from the scater R package (19, 20) and values were consequently log2 transformed. Principal component analysis (PCA) was performed on the 1,000 most variant genes with an average expression above one. The first 15 PCs were selected based on visual inspection of variance explained by each PC (scree plot). Cell heterogeneity was visualized by flt-SNE (21) using “max_iter = 2000” and default parameters. Cell clustering was performed with the *FindClusters* function from the Seurat R package, with a resolution of 0.3 and default settings. Marker genes for each cluster were identified by the *FindAllMarkers* function, using parameters “test.use = “roc”,” “min.pct = 0.25” and “logfc.threshold = 0.25.”

We applied the Monocle (version 2) algorithm (22) to determine the potential lineage differentiation between WT and VISTA KO monocyte populations. Cells in cluster 6 (granulocytes) were excluded from this analysis as they were not monocytes. Raw messenger RNA cell counts were used as input and a CellDataSet object was created with the parameter “expressionFamily = negbinomial.” Marker genes identified by the *FindAllMarkers* function were used as input. Default parameters were used in consequent steps to construct the monocyte differentiation trajectory.

### Chemokine Receptor, Consumption, Downmodulation, and Recycling

To address chemokine consumption, BMDMs were cultured in prewarmed RPMI-1640 medium (Hyclone, SH30027.01) containing either 0, 10, or 100 ng/ml CCL3 or CCL2. BMDMs were incubated at 37°C, 5% CO_2_ for 18 h and the supernatants subsequently removed and analyzed by ELISA (Thermofisher, Ready-Set-Go). For chemokine receptor downmodulation and recycling, thioglycolate-elicited peritoneal macrophages from WT and VISTA KO mice were seeded in prewarmed RPMI-1640 medium (Hyclone), supplemented with 10% FBS and 2 mM HEPES at a density of 2 million cells/ml in round-bottom polypropylene tubes (Corning) containing either 100 ng/ml CCL3, 100 ng/ml CCL2, or 500 ng/ml CCL5. Cells were incubated for the following times: 0, 2, 4, and 7 h. All recombinant chemokines were purchased from Peprotech. To investigate chemokine-induced recycling, cells were treated as described above for 2 h at 37°C, then were placed on ice and immediately washed twice in RPMI medium to remove free chemokines. Cells were then incubated at 37°C for 1 h to allow for receptor recycling and stained with antibodies directed against the various chemokine receptors assayed as described in flow cytometry and analysis.

### IHC

Tissues were fixed in 10% neutral buffered Formalin, paraffin embedded, and standard IHC was performed using anti mouse F4/80 (ab111101; Abcam, Cambridge, MA) at 1/100 dil. followed by incubation with goat anti rabbit IgG HRP secondary Ab (ThermoFisher 31462) at 1/3000 dil. Cy3.0 TSA Plus (Perkin Elmer, Waltham, MA) was employed for detection and the InForm software (Perkin Elmer) was utilized for quantitation of F4/80 in stained tissue sections.

### *In vitro* Chemotaxis Studies

*In vitro* migration assays were performed in media containing 0.5% FBS. For macrophage assays, a total of 2 × 10^5^ cells were applied to the upper chamber of 24-well plate HTS transwells with a pore size of 8 μm (Corning) and incubated for 2 h to adhere to the transwells at cell culture conditions. Lower chambers were set up with 200 ng/well of either CXCL13 (irrelevant chemokine control; Peprotech), CCL3 (Peprotech) or CCL5 (Peprotech). Plates were then incubated for 20.5 h, and then transwells were fixed with 4% paraformaldehyde (PFA) and stained with 0.1% (w/v) Crystal Violet (Millipore Sigma, St. Louis, MO, USA). Cells adhering to the top side of the transwell inserts were removed by gently scraping with a cotton swab applicator (Medline Industries, Mundelien, IL, USA). The inserts were then rinsed gently and allowed to air-dry overnight. Transwells were then visualized with an Olympus CK40 inverted microscope (Olympus Life Science, Tokyo, Japan) and photographed with a Hitachi 3CCD HV-C20 digital camera (Hitachi, Tokyo, Japan) at 50X magnification. Each condition was performed in duplicate wells. Fifteen photographs were taken per insert and transmigrated cells were quantified using the cell counting plugin of the FIJI program (open-source, available online at fiji.sc).

For MDSC assays, a total of 10^5^ cells were applied to the upper chamber of 5 μm HTS Transwell 96 well plates. Lower chambers were set up with a dose titration of either CXCL13 (irrelevant chemokine control, Peprotech) or CCL3 and plates were incubated for 12 h. Migrated cells were quantified by harvesting cells from the lower chamber for flow cytometry. Cells were stained with anti-CD11b and Gr-1 antibodies (Biolegend), and CD11b^+^ singlet events were quantified on a MACSQuant Analyzer 10 (Miltenyi Biotec). Data is presented from the dose of chemokine that induced the greatest migration in the WT controls.

Chemotactic indices were then calculated by dividing the mean number of cells for a given chemokine by the mean number of cells for the no-chemokine wells.

### *In vivo* MDSC Migration Studies

WT and VISTA KO MDSCs were dye-labeled with either CFSE or Violet CellTrace™ dyes (Thermofisher) at 1 μM concentration in PBS containing 0.1% BSA for 10 min at 37°C and then were washed three times in complete media. WT and KO cells were mixed at a 1:1 ratio. To ensure against dye-specific effects, experiments were repeated with the dye combination switched for WT vs. VISTA KO cells. Mixed dye-labeled WT and KO MDSCs were adoptively transferred i.v. into WT mice bearing tumors >10 mm in diameter. Each recipient mouse received 5 million WT and 5 million KO MDSCs. Twelve hours later, cells were isolated from the tumor with the Miltenyi tumor dissociation kit, and the spleen by homogenization through cell strainers. Tumor cells were enriched for MDSCs by staining with CD11b-PE (Biolegend) followed by enrichment for PE stained cells with a PE selection kit (StemCell Technologies). Finally, spleen and enriched tumor cells were stained for CD45, CD11b, and Gr1 as described above, and analyzed by flow cytometry. FACS analysis was performed to identify WT and KO MDSCs in the spleen vs. the tumor.

### Peritoneal Migration

Migration to the peritoneum was induced by i.p. injection of 1 μg of CCL2 in 250 μl of sterile saline. At 5 h post-injection, mice were euthanized and cells were isolated from the peritoneum. To increase consistency of cell numbers, 4 ml was collected after massage of a 5 ml PBS peritoneal injection. Cells were stained for infiltrating monocytes (CD11b^+^ Ly6C^hi^ Ly6G^−^) by flow cytometry.

### Statistical Analysis

Statistical analysis performed using GraphPad Prism. Data acquired with two variables (timecourses or dose titrations) was analyzed with 2-way ANOVAs followed by Sidak's multiple comparisons test. For comparisons between two groups, an un-paired *t*-test was performed. ^*^p ≤ 0.05, ^**^p ≤ 0.01, ^***^p ≤ 0.001, ^****^p ≤ 0.0001. Values of *p* ≤ 0.05 were considered significant.

## Results

**Figure 1:**
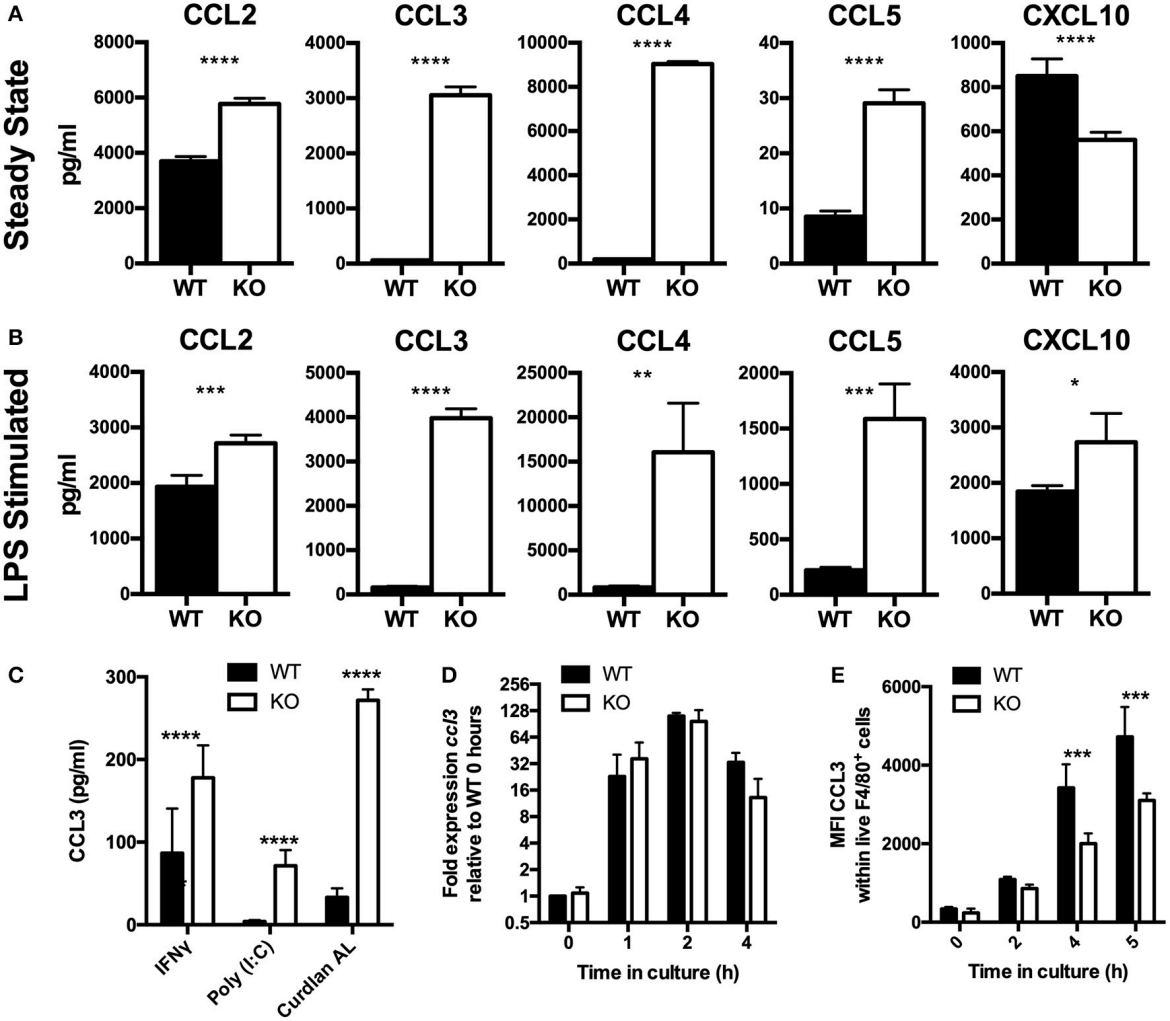
Chemokines accumulate to higher levels in VISTA KO supernatant, by a non-transcriptionally controlled mechanism. **(A–C)** Chemokines were measured in WT and KO BMDM culture supernatant by Luminex. **(A)** BMDMs were cultured for 48 h without stimulation. **(B)** BMDMs were stimulated *in vitro* with 10 ng/ml LPS for 24 h. **(C)** BMDMs were stimulated *in vitro* with 10 μg/ml Curdlan AL, 10 ng/ml Poly (I:C), or 10 ng/ml IFNγ for 24 h. **(D)** BMDMs were stimulated with 100 ng/ml LPS for the indicated time periods then gene expression was examined by qPCR. *Ccl3* expression is shown normalized to *Actb* as fold expression levels relative to the WT samples at T = 0 h. **(E)** BMDMs were stimulated with 100 ng/ml LPS for the indicated time periods and then stained for CCL3 by intracellular cytokine staining. Data shows MFI of CCL3 within total WT and KO macrophages. Data are representative of three independent experiments. *p ≤ 0.05, **p ≤ 0.01, ***p ≤ 0.001, ****p ≤ 0.0001.

The findings in this manuscript establish a pivotal role of VISTA in myeloid chemotaxis and mediator production. Although VISTA was initially described as an NCR of T cell biology, multiple studies suggest that VISTA also impacts on myeloid cell responses (9, 14, 23). However, none of these studies clearly defined a cell-intrinsic role for VISTA on myeloid cells. We therefore determined the impact of VISTA deficiency on cytokine and chemokine responses of murine macrophages. Analysis of a panel of cytokines and chemokines in the culture supernatant of macrophages from VISTA KO or WT mice revealed striking steady-state increases in the levels of a specific subset of inflammatory chemokines after 48 h (Figure 1A). In particular, the beta chemokines CCL2, CCL3, and CCL4 were profoundly upregulated while we observed a reduction in the chemokine CXCL10. Transcriptional profiling revealed that the chemokines were not altered at the mRNA level (Table S1). Furthermore, we showed a profound reduction in the chemotactic capabilities of VISTA-deficient macrophages to migrate both *in vitro* and *in vivo*.

Chemokines are among the earliest genes to be transcribed by migrating macrophages in response to inflammation and orchestrate important aspects of the inflammatory response (5, 24). Studies presented clearly established enhanced chemokine production by VISTA deficient macrophages under steady state conditions. We next tested if there was an impact of VISTA deficiency when macrophages were stimulated to produce chemokines by LPS. After 24 h, markedly higher levels of chemokines were detected in VISTA KO culture supernatants (Figure 1B), indicating that even strong induction of chemokines could not mask the phenotype imparted by VISTA deficiency. Furthermore, chemokine levels stimulated by interferon gamma (IFN-γ) or other TLR ligands was also enhanced in VISTA KO myeloid cells, indicating that the disparity was maintained under broad stimulation conditions (Figure 1C). In view of the fact that elevation in steady-state chemokines caused by VISTA deficiency was found to be non-transcriptionally controlled, we examined the production of chemokines over time focusing on the CCR2 ligand CCL2 and the CCR1, CCR3, and CCR5 ligand CCL3. The increased chemokine accumulation of CCL3 in culture supernatant occurred despite similar gene expression between WT and VISTA KO cells (Figure 1D), and decreased protein production (Figure 1E). Similar data was observed for CCL2 (data not shown).

**Figure 2:**
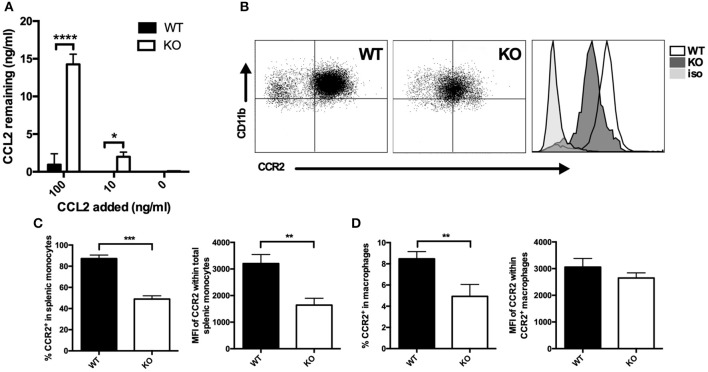
Membrane CCR2 expression is dysregulated on VISTA KO myeloid cells. **(A)** WT and KO thioglycolate-induced peritoneal macrophages were incubated with recombinant CCL2 for 18 h, and the concentration of the remaining chemokine was determined by ELISA. CCR2 expression was determined by flow cytometry on splenocytes **(B,C)** or peritoneal macrophages **(D)** from VISTA KO and WT mice. For **(C)**, percent CCR2 and MFI is shown within the total CD11b^+^ Ly6C^hi^ Ly6G^−^ pro-inflammatory monocyte population. In **(D)**, percent CCR2 is shown within F4/80^hi^ macrophages, and MFI is shown within the positive cell gate. Data are representative of three independent experiments. *p ≤ 0.05, **p ≤ 0.01, ***p ≤ 0.001, ****p ≤ 0.0001.

Since CCL2 and CCL3 protein expression as assessed by intracytoplasmic staining was not enhanced in VISTA KO macrophages despite the strikingly elevated levels of protein in the supernatant, we investigated whether the consumption of CCL2 was altered in the absence of VISTA. Macrophages were incubated with exogenous recombinant CCL2 (0, 10, and 100 ng/ml) for 18 h, a timepoint at which endogenous CCL2 does not accumulate to significant levels in either WT or KO cultures. The remaining concentration in the cell culture supernatant was then determined. WT macrophages were able to clear the vast majority of the chemokine when cultured in 100 ng/ml of exogenous CCL2, and the chemokine was undetectable when 10 ng/ml of CCL2 was added (Figure 2A). In contrast, at these concentrations of exogenous added chemokine, KO macrophages did not efficiently consume CCL2, and much higher levels were detected, with 13 ng/ml remaining after addition of 100 ng/ml, and 2 ng/ml remaining after culture with 10 ng/ml of CCL2 (Figure 2A). Since CCR2 is the primary receptor for CCL2 (25, 26), we assayed whether there were differences in the expression of this receptor between WT and VISTA KO cells. Indeed, there was a significant reduction of CCR2 surface expression on multiple myeloid subsets in the spleen, including macrophages and Inflammatory “Classical” monocytes; a population that expresses high CCR2 levels (Figures 2B–D). This disparity in receptor expression was also observed in bone marrow, indicating that this was not a tissue-specific effect (Figures S1A–E). Therefore, the enhanced accumulation of CCL2 appeared to be due to impaired consumption of the chemokine due to reduced receptor expression.

**Figure 3:**
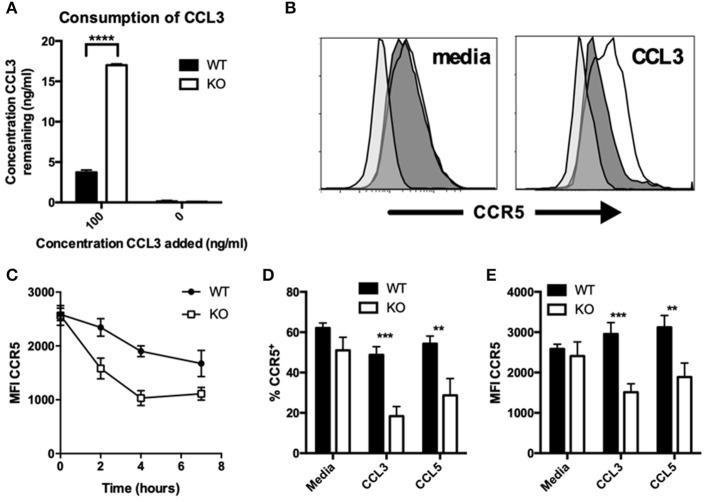
CCR5 recycling is dysregulated on KO macrophages after chemokine stimulation. **(A)** WT and KO peritoneal macrophages were incubated with indicated concentrations of recombinant CCL3 for 18 h, and the concentration of the remaining chemokine was determined by ELISA. Levels of CCR5 were assessed by flow cytometry on WT and KO peritoneal macrophages stimulated with and without 100 ng/ml CCL3 **(B–E)** or CCL5 **(D,E)** for 2 h **(B,D,E)** or the indicated timepoints **(C)** followed by 1 h for receptor recovery. Data are presented as overlays **(B)**, MFI **(C,E)**, or as percent of live singlet events **(D)**. Data are representative of two independent repeats. **p ≤ 0.01, ***p ≤ 0.001, ****p ≤ 0.0001.

In addition to CCL2, the consumption of CCL3 was also reduced in VISTA KO macrophages. Experiments were performed with both thioglycolate-elicited macrophages (Figure 3A) or BMDMs (Figure S1F) with similar results. We assessed the expression levels of the chemokine receptors for the chemokines CCL3, CCL4, and CCL5 at both steady-state and following chemokine-induced downmodulation. In contrast to CCR2, there were no significant differences in the surface levels of CCR5 between VISTA KO and WT macrophages at steady-state (Figures 3B–E). Only after treatment with CCL3 or CCL5 did the VISTA KO macrophages show markedly lower levels of CCR5 (>2.5 fold; Figures 3B–E). This effect was maintained at later time points following treatment (Figure 3C), and was not seen with the other relevant chemokine receptors CCR1, CCR3, or CCR4 (Figures S1G,H). These data indicate that VISTA is involved in the regulation of the surface expression of a subset of chemokine receptors, and altered chemokine receptor recycling appears to have a significant impact on chemokine accumulation. There are a litany of molecules that can regulate the activities of chemokine receptors and chemotactic activity. For example, multiple members of the tetraspanin family fine-tune the migratory activity vs. the antigen presentation activity of dendritic cells. CD81 and CD37 promote migration, while CD82 restrains migration. On the other hand, CD37 reduces while CD82 promotes APC activity. Synaptotagmins are another class of molecules that regulate chemotaxis but do not regulate chemokine or chemokine receptor expression (27). SYT7 and SYTL5 are positive regulators while SYT2 is a negative regulator of leukocyte migration. Synaptotagmins regulate leukocyte chemotaxis by linking chemoattractant-induced calcium flux to exocytosis and uropod release. However, to our knowledge, this is the first description of an NCR impacting on chemokine receptor recycling and chemokine production.

We next examined whether the altered chemokine receptor recycling or chemokine production was the result of differences in the development of myeloid precursors. The colony forming potential of WT vs. VISTA KO bone marrow for granulocytes was slightly but significantly higher in VISTA KO bone marrow, and was similar for all other subsets tested (Figure S2A). Further, the distribution of different myeloid precursors in WT or KO mice was similar, with only a minimal increase in early granulocyte/macrophage progenitors (Early GMP; cKit^+^ Ly6C^−^ CD115^−^; Figures S2B,C). Overall, these data suggest very small differences in bone marrow myeloid potential and no detectable difference in the generation of cells within the macrophage lineage. These data are important as it is the first to establish that the genetic deficiency of VISTA exerts no definable impact on myeloid lineage development. Given the differences we observed in CCR2 expression and monocyte migration, we sought to interrogate the impact of VISTA deficiency on cell-state signature, differentiation and heterogeneity of the monocyte population using single-cell RNA-seq. Monocytes were purified from CX_3_CR1-Cre VISTA deficient or WT control mice via FACS-sorting. Consistent with previously published data we observed multiple monocyte cell-states, which included the well-defined “Classical” Inflammatory Ly6C^hi^ CCR2^hi^ monocyte (Cluster 1) and Patrolling Ly6C^−^ CX_3_CR1^hi^ Nr4a1^+^ monocyte subsets (Cluster 2) [Figures S2D,G; (28–31)]. We also observed two clusters defined by high expression of MHC-II antigen presentation genes (Clusters 3 and 5) as well as a subset of the Inflammatory CCR2^hi^ monocytes defined by high IFN-I signature genes (Cluster 4) (Figures S2D,G). Of note, Cluster 5 had high expression of CD209a and MHC-II which is reminiscent of the phenotype of Monocyte-derived dendritic cell precursors recently defined (32). However, we did not note a significant difference in the abundance of monocyte populations in the spleens of WT vs. VISTA deficient mice (Figure S2E). Monocyte differentiation trajectory inference recapitulated the previously published monocyte lineage trajectory (33–37), starting with the Inflammatory monocytes which differentiate into the Patrolling subset (Figures S2F,H). Although this inference suggested that the Inflammatory monocyte subset (Cluster 1) bifurcated into an IFN-I^hi^ monocyte subset (Cluster 4) and the Patrolling monocyte subset (Cluster 2), we believe the Inflammatory IFN-I^hi^ monocytes (Cluster 4) are capable of differentiating into the Patrolling monocyte subset (Cluster 2), due to their high expression of *Ly6c* and *Ccr2* and low expression of *Cx3cr1* indicating immaturity (Figure S2G). However, we did not observe any differences in the differentiation between WT vs. VISTA KO monocytes and indeed report an identical differentiation trajectory (Figure S2F).

An alternative mechanism for the reduced chemoresponsiveness of VISTA-deficient myeloid cells could be a result of an alteration in the levels of proteases that affect the activity of these chemokines. The protease CD26/DPP IV can inhibit chemokine activity of multiple chemokines including CCL3 by provoking NH_2_-terminal proteolysis (38–42). We assessed the expression of CD26 at both the mRNA and protein levels, and found that it was not expressed by macrophages at either the transcriptional (Table S2) or protein levels (Figure S3A). Another mechanism by which chemokine responsiveness and cytokine production could be altered would be an indirect consequence of a change in the levels of adhesion molecules. As such, we assembled a comprehensive panel of adhesion molecules and examined whether any differences in the expression of these molecules exist between VISTA KO and WT macrophages. We found that VISTA deficiency had no significant impact on the expression of these molecules (Figure S3B). While transcriptional data points to the contrary, it remained possible that VISTA-deficiency altered the activation state of the macrophages, leading to changes in cytokine and chemokine levels. However, assessment of the phenotype of VISTA KO vs. WT macrophage revealed no significant differences in activation or M1/M2 markers (Figure S3C). Therefore, we present compelling evidence that these potential mechanisms do not contribute to the difference in chemokine production and chemotactic deficits in VISTA KO myeloid cells.

**Figure 4:**
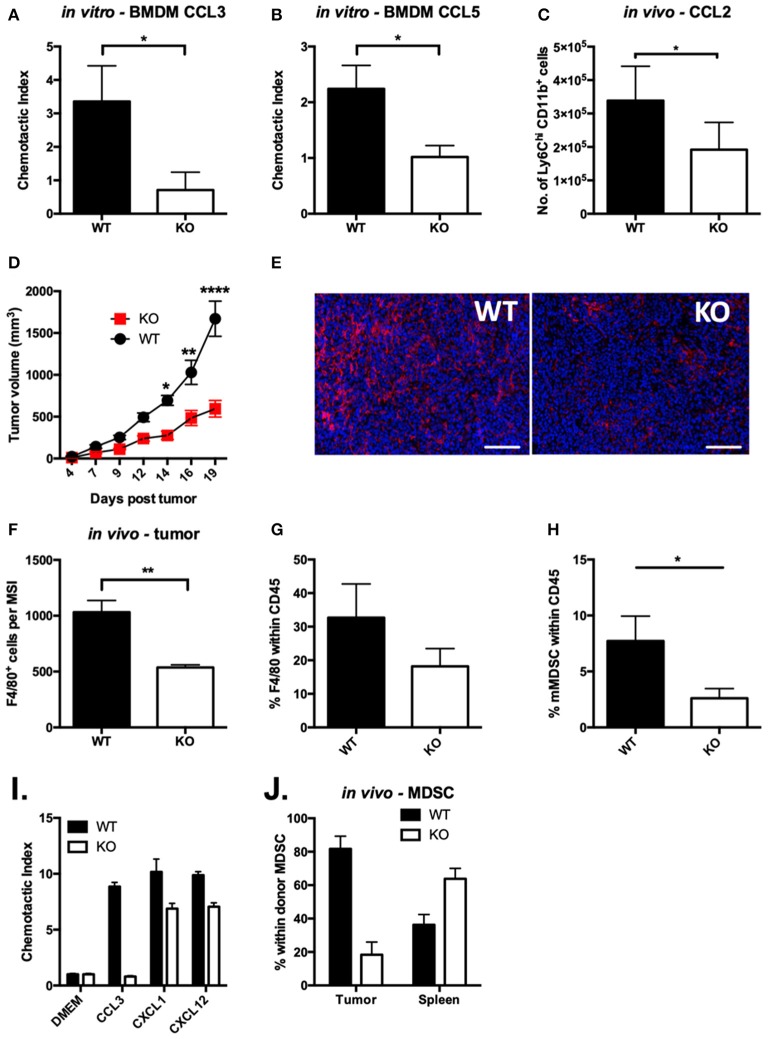
Chemotaxis of VISTA KO myeloid cells is defective *in vitro* and *in vivo. In vitro* transwell assays were performed using thioglycolate-induced peritoneal macrophages from WT and KO mice. The lower chamber contained 10 ng/ml of CCL3 **(A)** or CCL5 **(B)**. Cells that had migrated to the lower side of the membrane were stained with crystal violet after 18 h. Data shown are for duplicate wells pooled from three independent repeats. Data shown are the mean ± SD. **(C)** Migration to the peritoneum was induced by i.p. injection of 1 μg of CCL2 in WT and VISTA KO mice. At 5 h post-injection, peritoneal lavages were stained for infiltrating monocytes (CD11b^+^ Ly6C^hi^ Ly6G^−^) by flow cytometry. Data are representative of three independent experiments. **(D)** WT and KO mice were inoculated with 100 k CT26 cells and tumor size was monitored with digital calipers. **(E,F)** CT26 tumors in WT and KO mice of matched tumor volume were formalin-fixed, embedded and stained for F4/80 (red) and DAPI (blue). Scale bars indicate 100 μm. **(F)** Quantitation of 100 multi-spectral image (MSI) fields using the InForm software package. **(G,H)** Cells were isolated from CT26 tumors and stained by flow cytometry for myeloid cell populations. Plots show the percent of macrophages (**G**, CD11b^+^, F4/80^+^, Gr1^−^) and monocytic MDSCs (**H**, CD11b^+^,F4/80^+^, Gr1^−^) within live CD45^+^ events. **(I,J)** MDSCs isolated from spleens of WT or KO mice bearing CT26 tumors were dye labeled and mixed at a 1:1 ratio of WT (CFSE) to KO (violet) and then were tested for migration *in vitro* **(I)** or *in vivo* **(J)**. **(I)** MDSCs were placed in upper wells of 5 μm transwell plates and allowed to migrate to 5 ng/ml (25 ng/ml) per well of each of the indicated chemokines for 12 h. Cells that had migrated to the bottom chamber were counted by flow cytometry. **(J)** MDSC were adoptively transferred into CT26 tumor-bearing mice, and allowed to distribute for 12 h. Donor cells were identified in the CD11b-bead enriched tumor cells and spleen by flow cytometry. Migration experiments were performed three times. *p ≤ 0.05, **p ≤ 0.01, ****p ≤ 0.0001.

Our studies provide compelling evidence that VISTA KO macrophages are significantly less able to clear the chemokines CCL2 and CCL3 from solution. Both CCR5, a receptor for CCL3 and CCL5 (43), and CCR2, the primary receptor for CCL2 (44), have been shown to engage in chemokine scavenging to remove chemotactic trails as they are followed by migrating cells (45). The regulation of CCR2 and CCR5 surface expression at steady-state and in response to ligand, respectively, could impact chemokine abundance. To investigate the functional impact of the chemokine receptor dysregulation, we evaluated the chemotactic activity of VISTA KO myeloid cells. Chemotaxis to CCL3 and CCL5 was significantly altered in VISTA KO macrophages *in vitro* (Figures 4A,B). Chemotaxis of monocytes to CCL2 *in vivo* was also significantly reduced both in VISTA KO treated mice (Figure 4C). These data clearly show that VISTA deficiency selectively impairs the chemotactic response to specific chemokines.

VISTA is a potential immunotherapeutic target and its blockade reduces tumor growth (14, 46). This is associated with alteration of the tumor microenvironment and therefore we sought to determine if altered chemotaxis from VISTA deficiency may impact on tumor growth (7) or the composition of myeloid cells in the tumor microenvironment (TME). We employed the syngeneic colon cancer CT26 tumor model. By day 14 post-tumor inoculation, growth kinetics were significantly delayed in VISTA KO mice compared to WT mice (Figure 4D). At day 15, the composition of the immune infiltrate was examined in the tumors by immunohistochemistry (IHC) (Figures 4E,F). Strikingly, we observed much lower frequencies of F4/80^+^ tumor-associated macrophages (TAMs) in tumor sections and by flow cytometry on immune infiltrate derived from VISTA KO mice than in WT tumors (Figures 4F,G). Additionally, by flow cytometry we observed significantly reduced frequencies of monocytic MDSCs (Figure 4H). TAMs differentiate from monocytic MDSCs, so these data suggested a defect in MDSC migration. To directly evaluate if MDSC chemotaxis was altered by VISTA deficiency, we performed *in vitro* and *in vivo* migration assays. Similar to VISTA KO macrophages, VISTA KO MDSCs were defective in migration toward CCL3 across a transwell system (Figure 4I). Furthermore, when WT and KO MDSCs were adoptively co-transferred into untreated WT tumor-bearing mice, donor KO MDSCs infiltrated the tumor to much lower levels than WT MDSCs (Figure 4J). These data indicate a crucial impact of VISTA as a positive regulator of chemotaxis of multiple myeloid subsets.

## Discussion

This study presents multiple novel insights for the role of VISTA in the homeostatic regulation of myeloid cell chemotaxis toward a specific set of chemokine signals. In multiple systems involving monocytes, macrophages, and MDSCs, we demonstrated that VISTA deficiency results in a steady-state dysregulation of chemokine accumulation and chemotactic responses. This defect was not a consequence of transcriptional dysregulation of either chemokines or their cognate receptors, but due to differential consumption of the secreted chemokines. VISTA-deficient myeloid cells showed a marked dysregulation in the surface expression of chemokine receptors CCR2 and CCR5, and by consequence, enhanced accumulation of their ligands CCL2 and CCL3. As a result, the loss of VISTA resulted in a loss of chemotactic responses toward these chemokines.

Our studies provide compelling evidence that VISTA KO macrophages are significantly less able to clear the chemokines CCL2 and CCL3 from solution. Both CCR5 and CCR2 engage in chemokine scavenging to permit migration (45), to remove pro-inflammatory chemokine at the resolution of inflammation (43, 47), or as an anti-inflammatory mechanism mediated by IL-10 (48). Unlike CCR2, no differences were observed in steady-state expression levels of any of the CCL3 receptors between WT and VISTA KO macrophages. Steady-state surface levels of CCR1, CCR4, and CCR5 were identical in WT and VISTA KO macrophages as shown by flow cytometry analysis. Intriguingly, we found that CCR5 levels were nearly 2-fold lower in VISTA KO macrophages upon treatment with CCL3, suggesting a clear dysregulation of receptor downmodulation and/or recycling. The absence of steady-state differences in CCR5 indicates that the mechanisms for trafficking newly synthesized CCR5 to the membrane (49) are not impaired in the VISTA KO, implying that the defect in VISTA KO CCR5 levels subsequent to chemokine treatment is due to an inhibition of recycling rather than replacement with new protein. Recycling of chemokine receptors is regulated by a complicated, highly orchestrated array of signaling pathways, and post-translational modifications (50). Evolutionarily, CCR5 and CCR2 are much closer to each other than the other chemokine receptors that do not show the VISTA phenotype [CCR1, CCR3 and CCR4; (51)]. While the C-C receptors often share a significant degree of homology, CCR5, and CCR2 share the most at 75% homology (52). These VISTA regulated chemokine receptors may contain a conserved motif that a VISTA-induced signaling protein recognizes. The regulation of CCR2 and CCR5 surface expression at steady-state and in response to ligand, respectively, could impact chemokine abundance.

Several studies have demonstrated an important role for VISTA as a negative checkpoint regulator for both adaptive and innate immunity. However, insights into the cellular and molecular roles for this molecule remain very deficient. There is precedent in the literature for a role in regulation of lymphocyte chemotaxis by negative checkpoint regulators. Cytotoxic T-Lymphocyte Associated Protein 4 (CTLA-4) increases the migration of T cells, and overcomes the TCR-induced stop signal allowing synapses between T cells and APCs (53). PD-1 was recently demonstrated to regulate T follicular helper cell positioning and recruitment to the follicle by suppressing CXCR5 signaling and restricting CXCR3 upregulation (54). OX40 has been shown to induce CCL20 expression upon T cell activation by cognate antigen (55) as well as to play a role in the migration of high-affinity CD4^+^ T cells to B cell follicles (56). To our knowledge, this is the first report of a negative checkpoint regulator that is central to myeloid cell motility and migration.

This study also offers two important and intriguing observations. First, despite its expression on progenitors and hematopoietic stem cells, VISTA does not appear to play any detectable role in the differentiation of myeloid cells in the monocytic lineage. Single-cell RNA-seq analysis did not reveal significant differences in the abundance or cell state of different monocyte subsets between VISTA KO and WT mice. Second, VISTA deficiency exerted a chemotactic paralysis of both Classical (Ly6C^hi^ CCR2^hi^) monocytes and MDSCs (Figure 4); an inflammatory and suppressive population, respectively. This impact was also observed with thioglycolate-induced proinflammatory peritoneal macrophages (M1-like) and M-CSF induced BMDMs, which display a more M2-like cell state (Figure 3). In addition, monocytes, MDSCs, and macrophages represent different stages of myeloid differentiation. This indicates that the regulation of chemotactic response by VISTA extends beyond a single differentiation stage and strikingly, even impacts myeloid cells of opposing immunological functions.

We extended these observations to tumor biology where the muted responses toward CCL2 and CCL3 due to the loss of VISTA clearly predicted an alteration in the chemotaxis of monocytic cells to the tumor site and a change in the immune cell demography of the TME (57). Indeed, *in vivo* MDSC recruitment to the tumor site was ablated by VISTA deficiency, and as a result, the TME in VISTA KO mice was highly deficient in TAMs (Figure 4). Le Mercier et al. showed that VISTA blockade altered the TME composition through reduction of monocytic MDSCs and enhancement in tumor-infiltrating lymphocytes (TILs) (14). These observations support the insights the current study elucidates about the impact of VISTA on tumor biology. Macrophages in tumors display consistent VISTA expression and therefore, we expect VISTA blockade to have broad implications on TAM recruitment and suppressive function (58). Accumulating evidence supports a central role for VISTA in the inflammatory response (6, 59). Myeloid cell migration by VISTA is also expected to be important for the outcome of inflammation, as has been recently demonstrated (9).

Single-cell analysis of splenic monocytes revealed no detectable impact for the loss of VISTA on monocyte heterogeneity or differentiation trajectory (Figure S2). However, to our knowledge, this is the first published report of splenic murine monocyte heterogeneity and cell-state by scRNA-seq. Inflammatory “Classical” monocytes had a distinct subset with an enriched IFN-I gene signature (Cluster 4). Indeed, we uncovered two clusters of MHC-II^hi^ monocytes. One of these clusters (Cluster 5) had high expression of CD209a and MHC-II and a transcriptional phenotype similar to monocyte-derived DC precursors. On the other hand, cluster 2 was defined as Ly6C^+^ CD43^+^ intermediate monocytes (60, 61). Human but not murine intermediate monocytes have been defined as HLA-DR^hi^ cells, and indeed our data shows that murine intermediate monocytes also are MHC-II^hi^.

We present VISTA as a novel checkpoint regulator that uniquely plays a central role in regulating chemokine and chemokine receptor responsiveness in myeloid cells at the earliest phase of the immune response. The current study unambiguously demonstrates an *in vitro* and *in vivo* chemotaxis deficit of both myeloid cells in the absence of VISTA, confirming the fundamental role of this immunomodulatory molecule in controlling the chemotactic behavior of leukocytes. Elucidating mechanisms of VISTA activities in immunity should aid in both understanding its immunoregulatory functions and may be of potential benefit in guiding novel strategies for immunotherapy.

## Data Availability Statement

The raw data supporting the conclusions of this manuscript will be made available by the authors, without undue reservation, to any qualified researcher.

## Ethics Statement

The animal study was reviewed and approved by Institutional Animal Care and Use Committee of Dartmouth College, NH, USA (protocols 2012 and 2014).

## Author Contributions

TB, ME, JL, RM, AS, WC, JD, EN, JLL, NS, and AK performed experiments. ES performed computational analysis. TB, ME, RN, and JLL conceived the study, designed experiments, and wrote the manuscript.

### Conflict of Interest

RN is co-founder of ImmuNext involved in commercial development of anti-VISTA antibodies. The remaining authors declare that the research was conducted in the absence of any commercial or financial relationships that could be construed as a potential conflict of interest.
