# Supplementary material for: Defining the Signature of VISTA on Myeloid Cell Chemokine Responsiveness
Source: Front Immunol. 2019 Nov 19;10:2641. doi: 10.3389/fimmu.2019.02641 (PMC6877598; doi:10.3389/fimmu.2019.02641)
Supplement: Supplementary file 1 [file Data_Sheet_1.pdf]

Supplementary Material

1. Supplementary Figures

Supplemental Figure S1. Chemokine receptor expression in response to CCL2 and CCL3 stimulation.

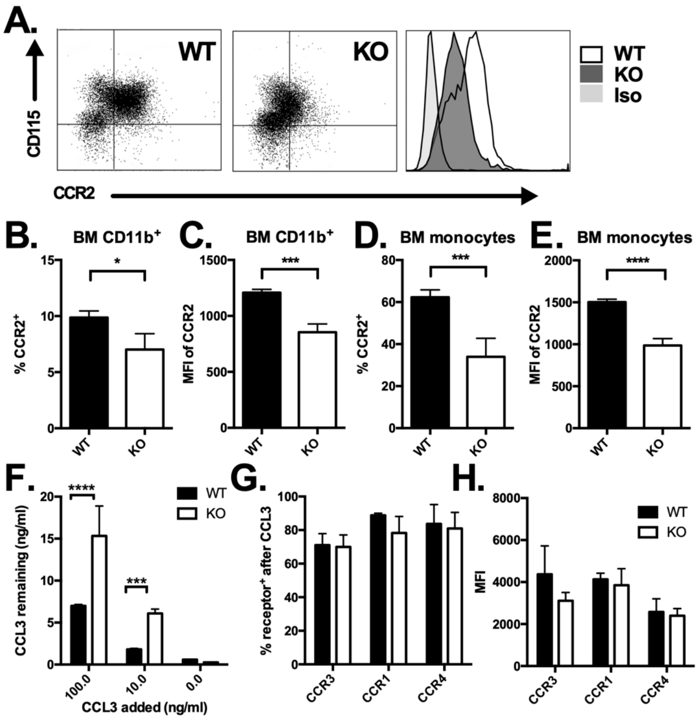

CCR2 expression was determined by flow cytometry on bone marrow from VISTA KO and WT mice. Cells are gated on total CD11b$^+$ myeloid cells (A to C) or CD11b$^+$ Ly6C^{hi} Ly6G$^-$ pro-inflammatory monocytes (D and E). A shows a representative scatter plot. B and D display data as percent positive relative to isotype controls, while C and E show MFI. Data are representative of three independent experiments. F) WT and KO BMDMs were incubated with indicated concentrations of recombinant CCL3 for 18h, and the concentration of the remaining chemokine was determined by ELISA. G and H) Levels of CCR3, CCR1, and CCR4 were assessed by flow cytometry on WT and KO peritoneal macrophages after treatment with 100 ng/ml CCL3 for 2h followed by 1h for receptor recovery. Flow data shown are representative of two independent repeats for CCR3, CCR1 and CCR4 and greater than three times for CCR5. Data is displayed as percent of F4/80$^+$ live cells (G) or as MFI (H), and is representative of three independent repeats.

Supplemental Figure S2: Myeloid cell development is normal in KO mice

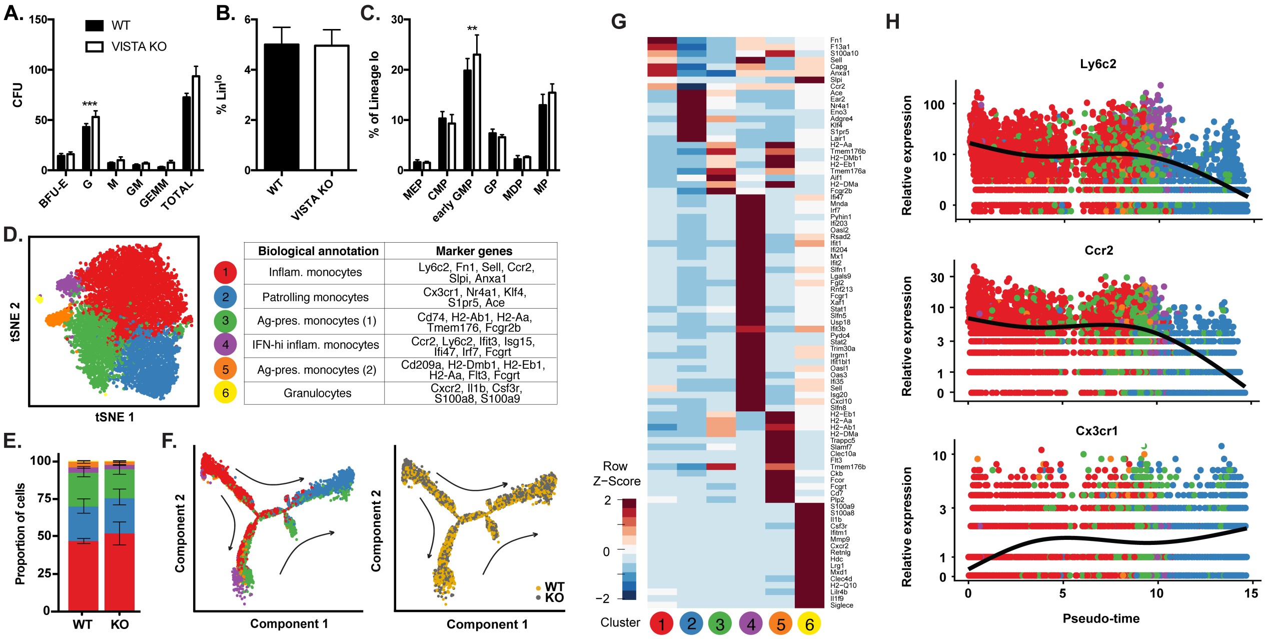

(A) Bone marrow (BM) from WT or KO mice was flushed and cultured in MethoCult GF M3434 medium for 12 days. Colonies were typed and counted for CFU per 10^4 BM cells. (B, C) Bone marrow from WT or KO mice was flushed and stained by flow cytometry for total lineage negative cells (B) and individual precursor subsets (C). Myeloid precursor lineages are defined as megakaryocyte-erythrocyte progenitors (MEP; $cKit^+$ $Sca1^-$ $CD16/32^-$), common myeloid progenitors (CMP; $cKit^+$ $Sca1^-$ $CD16/32^{lo}$), Early granulocyte/macrophage progenitors (Early GMP; $cKit^+$ $Ly6C^-$ $CD115^-$); granulocyte progenitor (GP; $cKit^+$ $Ly6C^+$ $CD115^-$), macrophage and dendritic cell progenitors (MDP; $cKit^+$ $Sca1^-$ $CD16/32^{hi}$ $CD115^+$), monocyte progenitors (MP; $cKit^+$ $Ly6C^+$ $CD115^+$). Experiments were performed three times. (D) Flt-SNE projection of WT and VISTA KO splenic monocytes (n=3 in each group), showing 6 main clusters. Clusters include two inflammatory (inflam.) monocyte clusters, a patrolling monocyte cluster, two antigen-presenting (Ag-pres.) clusters, and a granulocyte cluster, based on marker gene expression. Each dot corresponds to one single cell, colored according to cell cluster. (E) Bar graph of the proportion of cells in each cluster, separated by WT and VISTA KO groups (n=3). (F) Cell trajectory of clusters 1-5 inferred by Monocle 2, based on cluster or WT or VISTA KO origin. G) Heat map of the signature genes for each cluster and their differential expression across the clusters. H) Monocyte differentiation trajectory inference of monocyte differentiation showing the expression of Ly6C, CX_3CR1 and CCR2 which define the cell trajectory of Inflammatory monocytes towards Patrolling monocytes.

Supplemental Figure S3: The phenotype and adhesion molecule expression of VISTA KO macrophages is similar to WT.

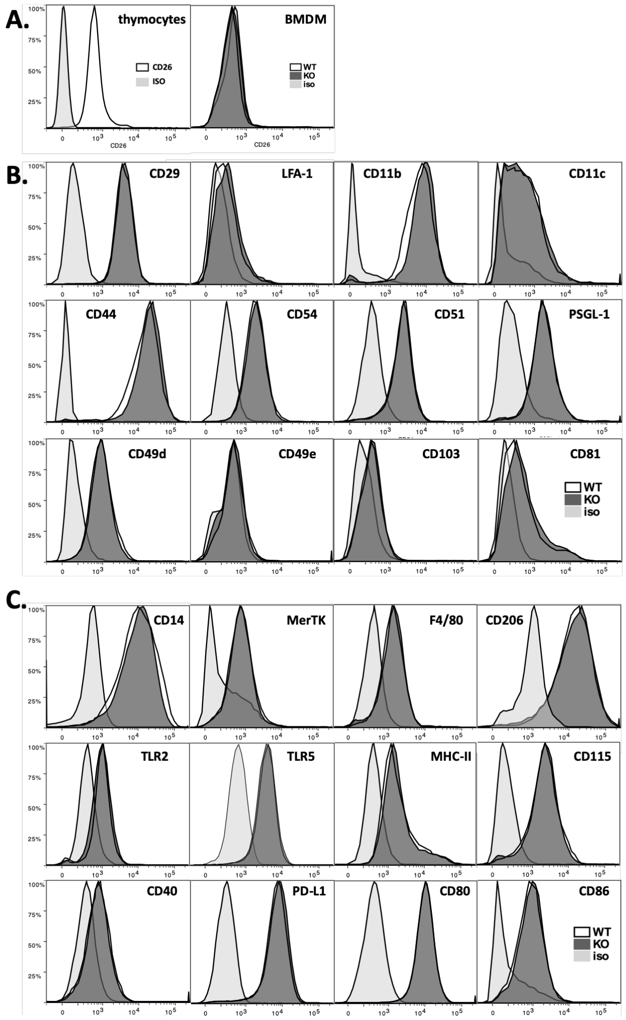

A) Expression of CD26 was determined by flow cytometry on WT and VISTA KO macrophages and compared to WT thymocytes as a positive control for staining. **B)** Expression of multiple adhesion molecules was determined by flow cytometry on WT and VISTA KO macrophages. **C)** Expression of multiple phenotypic and activation markers was determined by flow cytometry on WT and VISTA KO peritoneal macrophages. Data are representative of three independent experiments each with 4-5 replicate mice per group.

2. Supplementary Tables

Supplemental Table S1: Nanostring of Chemokine and Chemokine Receptors

WT and VISTA KO BMDMs were washed with fresh media, cultured for 6 hours, and then chemokine and chemokine receptor gene expression was examined by nanostring. The experiment was repeated twice, with 3-4 replicate mice and 2 well replicates.

Supplemental Table S2: Nanostring of Adhesion molecules.

WT and VISTA KO BMDMs were washed with fresh media, cultured for 6 hours, and then adhesion molecule gene expression was examined by nanostring. The experiment was repeated twice, with 3-4 replicate mice and 2 well replicates.
